# Application of Multi-Objective Optimization to Pooled Experiments of Next Generation Sequencing for Detection of Rare Mutations

**DOI:** 10.1371/journal.pone.0104992

**Published:** 2014-09-02

**Authors:** Julius Žilinskas, Algirdas Lančinskas, Mario Rosario Guarracino

**Affiliations:** 1 Institute of Mathematics and Informatics, Vilnius University, Vilnius, Lithuania; 2 High Performance Computing and Networking Institute, National Research Council, Naples, Italy; Institute of Genetics and Biophysics, Italy

## Abstract

In this paper we propose some mathematical models to plan a Next Generation Sequencing experiment to detect rare mutations in pools of patients. A mathematical optimization problem is formulated for optimal pooling, with respect to minimization of the experiment cost. Then, two different strategies to replicate patients in pools are proposed, which have the advantage to decrease the overall costs. Finally, a multi-objective optimization formulation is proposed, where the trade-off between the probability to detect a mutation and overall costs is taken into account. The proposed solutions are devised in pursuance of the following advantages: (i) the solution guarantees mutations are detectable in the experimental setting, and (ii) the cost of the NGS experiment and its biological validation using Sanger sequencing is minimized. Simulations show replicating pools can decrease overall experimental cost, thus making pooling an interesting option.

## Introduction

In the 1990s it became clear it was not possible to sequence all or large parts of the human genome using already available technologies. Indeed, sequencing the 3.6 billions base pairs using sequences shorter than one thousand base pairs would have been expensive and time demanding. Therefore, the problem was solved concurrently, dividing the long DNA sequence in to sub-sequences, and analyzing them in parallel.

The Next Generation Sequencing (NGS) technologies use this idea, producing a large number (in the order of ten of millions) of *short reads*, each being the result of the sequencing of a DNA fragment. These fragments are obtained firstly breaking the original DNA sequence, using ultrasounds or restriction enzymes, in pieces of a fixed size, and then cloning them in a process known as *amplification*. The result of this process is then sequenced, producing the short reads, whose length, at the moment of writing, varies between 50 and 500 base pairs, depending on the technology used by the sequencer. These nucleotide string sequences are stored in flat files, together with information on the quality of each base call. Then, each short read is aligned on the reference genome. The number of reads that aligns on a specific position is called *coverage*. The result of the alignment is a file containing the information about the alignment, such as the position on the reference genome where the short read has been mapped and its error probability, and the potential insertions or deletions needed to obtain the alignment. The data are then analyzed to detect variations with respect to the reference genome.

An example of mutation is a Single Nucleotide Polymorphism (SNP), where the mutation consists in a change of a single nucleotide into another. This can be detected in a certain position, looking at the nucleotides in each short read that are aligned with the position of interest. If the SNP is only on one allele (heterozygous mutation), then a half of the short reads might be different from the reference. Some of these polymorphisms are due to the natural variability of organisms, and some might be unique for a specific individual. Some of those mutations in protein coding genes can alter the associated protein, whereas some other have no effect, due for example to the degeneracy of genetic code. In case of rare genetic disease studies, we are interested in detecting mutations in the DNA sequence, that are unique for an individual and causative of the disease. Due to its rarity and complexity of its treat, these mutations might appear only in one patient in the population sample under study.

The widespread availability of high-throughput sequencing technologies makes it possible to analyze large panels of patients affected by rare genetic diseases, although the time and cost of such experiments are still a limiting factor. The existing solutions rely, when possible, in the analysis of pools of patients. This pooling technique consists in analyzing a mixture of DNA from a group of individuals, and assigning the discovered causative mutations to a single patient. The inspiration for the present work comes from a study conducted on muscular diseases, whose aim is to analyze the samples from a thousand patients affected by muscular diseases. To this extent, the sequencing experiment has been planned targeting a panel of genes taken from a previous study, where all known genes related to muscular diseases have been reviewed [1], and using Agilent HaloPlex Target Enrichment kit.

Examples of studies using pooled experiments, in various genetic and genomic applications, can be found in [2]–[4]. The limit of such studies is that no evaluation has been done to asses the group size of the pools, the probability to detect a mutation, the cost associated with the experiment and its biological validation.

When dealing with pooled DNA sequencing experiments, there are various aspects that need to be considered. First, the contribution of DNA from each patient in a pool is in general different. This is due to the fact that this operation is usually manually executed, and even a robot or a skilled professional biologist might produce a mixture in which the most represented individual contributes with a quantity of DNA that is double or more, with respect to the less represented one. Therefore, the number of short reads produced during sequencing and belonging to each patient might be very different. Since a certain percentage of reads is affected by base call and mapping errors, in the worst case, all mutations of a specific patient might be in reads that are unusable and therefore discarded from the analysis. For this reason, a rule of thumb used by biologists dealing with this kind of experiments is that the number of short reads covering each position of interest should be at least thirty. Such number should be multiplied by the number of patients in the pool, thus liming the maximum number of patients 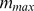 per pool, due to the limited coverage capability of sequencing methods.

Supposing that the disease has a complex genetic trait and is rare, we might expect each patient to bring a certain number of unique mutations, that will be detectable only in the short reads belonging to that specific patient. Therefore, there will be a certain number of mutations that will appear in the experiment. If we want to attribute these mutations to a specific patient, we need to test all patients for all found mutations, using for example low throughput Sanger sequencing technology. Although Sanger tests are orders of magnitude cheaper than the NGS ones, even when dealing with a low number of expected mutations in a pool of patients, this might result in a large number of experiments with a cost comparable with NGS. We will show that in some cases, it might be convenient to explore some controlling strategies that replicate patients in pools used in the high throughput experiment, in such a way that the number of Sanger sequencing experiments is decreased (or even eliminated), together with the total cost of the experiments. This will be achieved sequencing each patient twice, and allocating the patient in two different pools, which we call *main* and *replicated* pools.

In this paper we propose a technique to plan NGS pooled experiments. The solution takes into account experimental setups with or without replication of the patients. A combinatorial organization of pools has been devised with some optimality characteristics, whose advantages are: (i) the solution guarantees that the mutation is detectable in the experimental setting, and (ii) the cost of the experiment and its biological validations using Sanger sequencing is minimized. Then we use a probabilistic model to determine the number of patients for each pool, and a multi-objective optimization formulation of the problem to obtain optimal grouping of patients with a given probability to detect a mutation.

## Methods

We recall that when patients are organized in pools and analyzed with NGS technology, the assignment of found mutations to patients is accomplished using Sanger sequencing technology. In the following we provide a mathematical formulation of the problem, together with an analysis of results in terms of costs associated to the optimal allocation of patients in pools.

### Pooling without Replica

Suppose there are 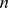 patients each of which might bring at most 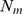 rare mutations. All 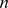 patients can be allocated in 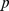 pools consisting of 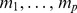 patients, such as:
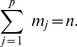
(1)


The size of each pool is restricted by a maximum number 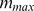 of patients, as explained in the previous section, therefore:
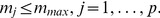
(2)


If 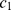 and 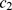 are the costs of NGS and Sanger sequencing, respectively, then the total cost of the experiment can be calculated by:
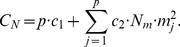
(3)


The total cost 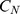 of the experiment depends on the cost 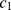 for sequencing each of the 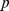 of pools, and the cost 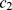 of the Sanger validations. Once 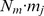 mutations have been found in the 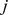-th pool, they need to be assigned to one of the 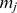 patients, thus requiring 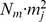 validations.

### Pooling with Replica

The validation of a biological experiment is usually obtained replicating the experiment at least twice. In this section we investigate two different strategies for pooled experiments. Both strategies are based on construction of pools so that (*i*) each patient is replicated two times in different pools, and (*ii*) if a mutation is identified in both main and replicated pool, it refers to at most 1 patient.

#### Transposition

**Figure 1:**
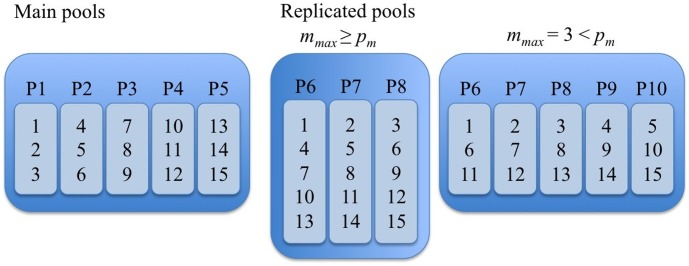
Illustration of allocation of patients in main pools (left), and in control pools, when 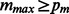 (center) and 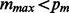 (right), using transposition strategy.

The first strategy, called Transposition, is based on the concept of matrix transposition as it is illustrated in the left and center panels of Figure 1, where the left image illustrates the set of 15 patients distributed into 5 main pools, and the middle image represents the replicated pools. Naturally this approach can be applied if the maximum number of patients allowed in a single pool is greater than or equal to the number of main pools (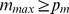). If the latter requirement is not satisfied (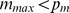), then the number of patients in a control pool would exceed the maximum number of patients 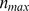 in a pool. Therefore a larger number of control pools must be constructed using a similar strategy, as it is shown in the right panel of Figure 1, in order to correctly fit all patients in replicated pools. In general, the number of replicated pools (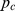) can be chosen by taking into account the following two constraints:

- the number of replicated pools cannot be larger than the total number of patients (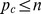), in order to have enough patients to complete all pools;
- the number of replicated pools cannot be smaller than the number of patients in the largest main pool: 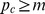, where
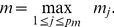
(4)

The second constraint is based on the requirement that all patients from a single main pool must be allocated in separate replicated pools, thus ensuring that identification of a SNP in any two pools would refer to at most 1 patient. Therefore, a SNP, detected in one main and one replicated pools, can be assigned to a single patient. Since no Sanger tests are needed, the price of pooling for this strategy can be expressed as
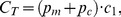
(5)where 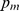 is the number of main pools, 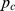 is the number of replicated pools, and 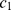 the price for NGS for each pool.

#### OptReplica

The second strategy, called OptReplica, is built so that for a given number 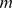 of patients in the largest pool the smallest number 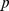 of pools is chosen subject to:
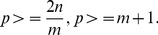
(6)


The first inequality is based on the requirement to fit all allocations of 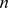 patients repeating each of them twice. The second inequality is based on the requirement to discriminate the SNPs belonging to any patient: identification in any two pools of a SNP must match at most 1 patient. So if 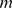 patients are allocated to the first pool, 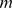 other pools are needed with each of these patients allocated to different pools, so that no two or more patients belonging to the same main pool are together in any other pool.

In the case of 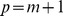, allocation of patients to pools correspond to 2-combinations of pools. Therefore the maximum number of patients to allocate is
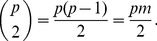
(7)


**Figure 2:**
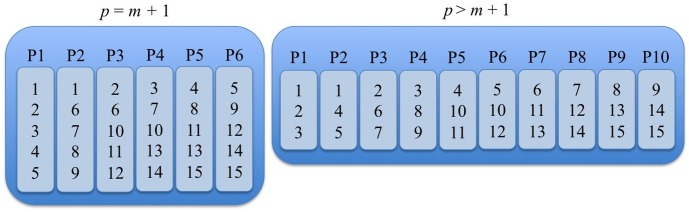
Illustration of allocation of patients in control pools using strategy OptReplica, when 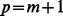 (left) and 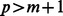 (right).

The algorithm to define allocations can be described as: *allocate each patient in both the first pool that is not yet completely filled, and in the first pool with the smallest number of allocated patients.* The same strategy can be used when 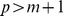. Examples are shown in Figure 2.

Since no Sanger tests are needed, the price of pooling for this strategy is
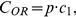
(8)where 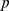 stands for the number of pools, and 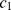 for the price for NGS of each pool.

#### Grouping of Patients

Both strategies Transposition and OptReplica allow identification of an SNP belonging to a single patient without any Sanger test. Naturally, the total cost of the experiment depends on the total number of pools, used for the allocation of the patients, and the lowest price can be obtained when the number of pools is smallest (see (5) and (8)). However, the lowest number of pools is restricted by the maximum number of patients (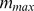) allowed in a single pool.

For example, if we use Transposition strategy for allocation of 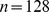 patients with 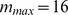, it would be optimal to use 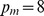 main pools with 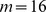 patients each. Since the number of replicated pools 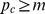, this would lead to 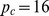 replicated pools, each with 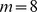 patients, thus resulting in half loaded replicated pools. The number of these pools can be reduced by aggregating patients into groups, each of which is considered as a single unit. For example, if 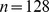 patients are grouped into 64 groups with 2 patients per group, then an optimal number 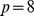 of replicated pools can be used. In this case, when a variation is found in both the main and replicated pools, the grouping will lead to an indecision between the two patients belonging to the group present in both pools, that need to be resolved with a Sanger sequencing.

**Figure 3:**
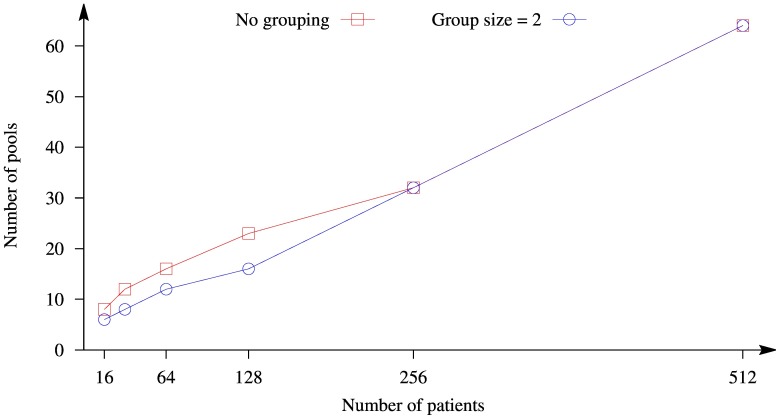
Dependence of the minimum number of pools on the total number of patients using Transposition strategy with 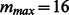.

The dependence of the minimum number of pools on the total number of patients using Transposition strategy is illustrated in Figure 3, where the horizontal axis corresponds to the total number of patients, the vertical axis to the number of pools, and different curves to the different sizes of groups: no grouping and with groups size equals 2.

On one hand, the grouping of patients can reduce the number of pools, on the other hand it leads to an identification of a group of patients instead of a single one. Therefore, Sanger tests must be additionally used to solve the indecision between patients in an identified group. The total cost of the experiment with grouping of patients can be expressed as:
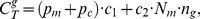
(9)where 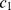 and 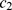 stand for the cost of NGS and Sanger tests respectively, 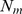 stands for the number of rare mutations per patients, and 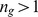 is the number of patients in a group.

### Multi-objective Optimization

The maximum number 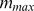 of patients allowed in a single pool may be determined with respect to the unity probability 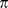 to detect the mutation. If the number of patients in a pool is lower than or equal to 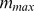, then 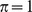; otherwise, if the number of patients in a pool exceeds the value of 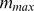 then the probability to detect the mutation becomes lower than 1, and decreases when the number of patients in a pool increases. This is due to the fact that for increasing number of patients, the number of reads representing DNA strings of a single patients decreases. However, the total cost of the sequencing can be notably reduced increasing the number of patients in a single pool. Therefore two conflicting objectives can be envisaged.

In order to analyze the influence of sequencing errors in the planning of NGS pooled experiments with respect to the total price of the experiment, a multi-objective optimization problem should be formulated by taking into account the following two objectives:

- minimize the cost 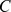 of sequencing, and
- maximize the probability 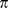 to detect the mutation.

It is assumed that the probability 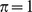 if the number (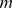) of patients in the largest pool is not greater than the maximum number allowed (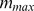), and 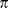 is zero if 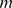 is greater than a given number 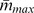. If the number of patients in the largest pool increases from 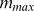 to 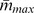, then the probability 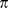 decreases from 1 to 0. The value of probability 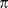 can be expressed as
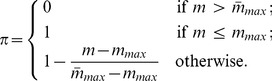
(10)


When objectives are conflicting, usually it is impossible to find a single solution, which would be the best according to both objectives. The solution which is the best by one objective can be worse, or even the worst, by another objective [5]. In such a case it is usual to find a set of compromising solutions, which are all optimal in some sense, thus providing to decision maker an opportunity to make a choice.

In general, two different solutions can be related to each other by the dominance relation. It is said that solution 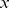 dominates solution 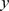 (denoted by 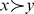) if, and only if [6]

- solution 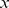 is not worse than solution 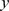 according to all objectives, and
- solution 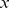 is strictly better than solution 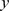 according to at least one objective.

If 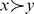 then solution 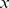 is called *dominator* of solution 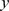. A solution with no dominators is called *non-dominated* (or *Pareto-optimal*), and the set of such solutions is called *Pareto set* [7]. The corresponding set in the objective space (values of the objective functions) is called *Pareto front*. For more detailed analysis of the Pareto optimality, reader can refer to [8]–[10].

**Figure 4:**
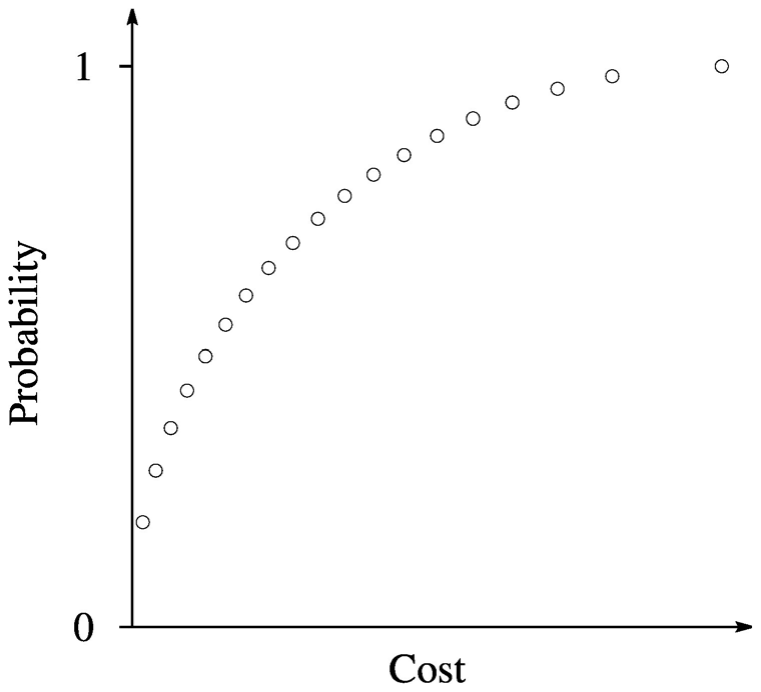
Illustration of Pareto front.

Since we deal with two objectives one of which is subject to minimize (the cost) and another – to maximize (the probability), the shape of Pareto front of the problem should be similar to one illustrated in Figure 4. One can see in the figure, the solution corresponding to the largest probability, corresponds to the largest cost of the experiment as well. On the other side the solution corresponding to the cheapest experiment, corresponds to the lowest probability as well. However it is possible to find a set of intermediate solutions which are all optimal in Pareto sense, and decision maker can choose the most appropriate one, taking into account various considerations, such as the given budget or/and the desired probability to detect the mutations, in our case.

## Results

### Experiments on Pooling without Replica

The first experiment is aimed at comparing the cost of NGS pooled experiments without replica. It is expected to detect 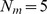 rare mutations per patient, considering the cost of NGS sequencing 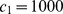 per pool, and 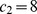 the cost of each Sanger sequencing to check whether a found mutation belongs to a patient. This ratio between costs is only indicative, and subject to vary over time. The total number of patients is chosen to be 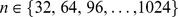, and the maximum number of patients in a pool is assumed to be 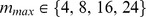. For each value of 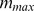 the lowest number of pools is selected so that the maximum number of patients in a pool would not exceed:
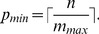
(11)


It is assumed that pools are composed by patients so that the difference between the largest and the lowest pools would be minimal, i.e. not greater than 1; e.g. if 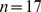 and 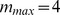, then 5 pools should be used, where 2 of them consist of 4 patients, and the remaining 3 of 3 patients. The results, obtained using 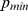 pools, are compared with the results, obtained using the optimal number of pools (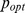), which is determined by the complete enumeration with respect to minimization of the cost:
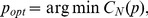
(12)where:
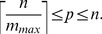
(13)


**Figure 5:**
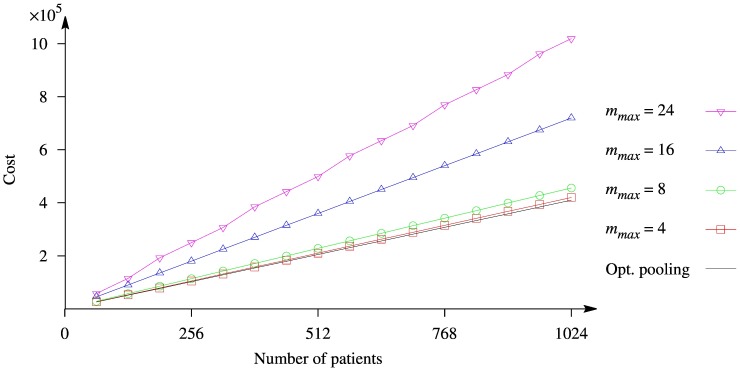
The cost of the sequencing versus the number of patients when allocating the maximum number 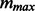 of patients per pool without any replica.

Results of the experiment are presented in Figure 5, where the horizontal axis corresponds to the number of patients and the vertical one to the cost of the pooled experiment. Different curves correspond to the different value of 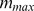, for which a minimal number 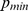 of pools is chosen, except the continuous curve (Opt. pooling), where the optimal number 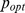 of pools is determined. The figure shows that the total cost of the sequencing increases together with the increment of the maximum number of patients in a pool, when the lowest number of pools (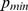) is used. However the lowest cost of the sequencing is obtained using the optimal number (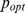) of pools, as it is illustrated by the black curve in Figure 5. The results, obtained using optimal values of 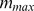, are the same as those obtained with 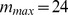, except the case of 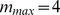 when the cost of the sequencing is slightly higher. This can be explained by the numerical results of the experiment, performed with 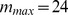. The optimal numbers 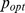 of pools and the respective optimal numbers 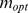 of patients in a pool using different numbers of patients are presented in Table 1 together with the number of Sanger tests needed and the total cost of the sequencing. The table shows that the optimal number of pools increases when the total number of patients increases, however the optimal number of patients in a pool remains almost stable and very much less than the maximum number of patients in a pool (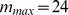).

**Table 1:** Cost of experiments and total number of Sanger tests needed to detect 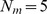 mutations in each patient, when using optimal number 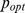 of pools without replica, and 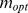 patients in a pool, for a fixed total number 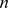 of patients.

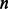	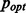	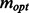	Number of Sanger tests	Cost
128	26	5	3160	51280
256	51	6	6430	102440
512	102	6	12860	204880
1024	205	5	25580	409640

### Experiments on Pooling with Replica

**Figure 6:**
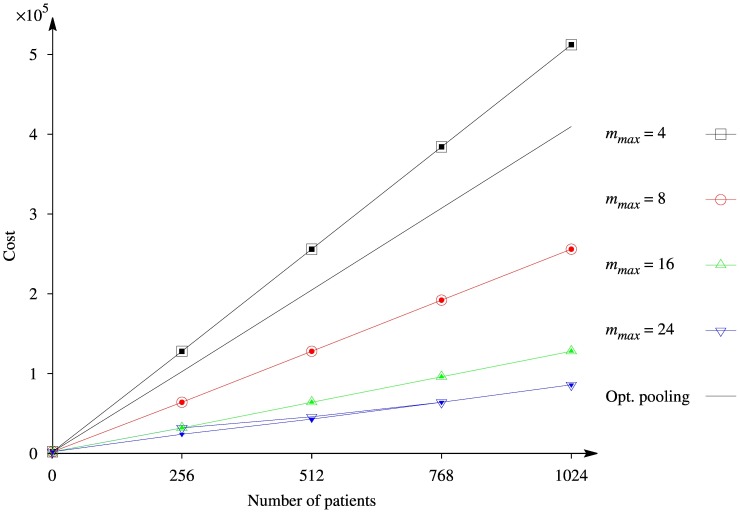
Comparison of the cost of NGS pooled experiment using different values of 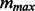 and different strategies of pooling: Transposition (hollow marks) and OptReplica (filled marks).

The next experiment is aimed at comparing the cost of the sequencing using two replica strategies, which lead to the sequencing without any indecision or indecision between a group of patients. The same values of 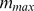 are used as they were in the previous computational experiment. The complete enumeration is used to determine the optimal numbers of main (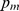) and control (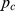) pools for Transposition strategy (see (5)), whereas the lowest number 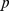 such as 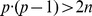 of pools is chosen for OptReplica strategy (see (8)). The results are presented in Figure 6, where the hollow marks stand for the cost obtained using Transposition strategy and the filled marks for the cost obtained by OptReplica; different curves correspond to different value of 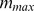. The line without marks stands for the cost of the sequencing obtained with pooling without replica into pools of optimal size.

The figure illustrates that the costs of the sequencing decrease when the maximum number of patients in a pool increases, independent whether Transposition or OptReplica strategy is used. The slight difference in cost between the strategies can be distinguished when a small number of patients are pooled into small pools and the OptReplica is then slightly better. It is also clear from the figure that usage of a replication decreases the cost of the sequencing.

The impact of grouping of patients to the total cost of the sequencing using Transposition strategy is illustrated in Table 2, where the first column is the total number of patients, the second column is the cost of the sequencing without grouping of patients, and the last column is the cost of the sequencing, when patients are grouped into groups of 2. One can see in the table that it is useful to use the grouping of patients if the number of patients is small (16 and 32).

**Table 2:** Impact of grouping of the patients to the total cost of the sequencing with replication using Transposition strategy.

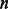	No grouping	Grouping by 2
16	8000	7280
32	12000	10560
64	16000	17120
128	23000	26240

**Figure 7:**
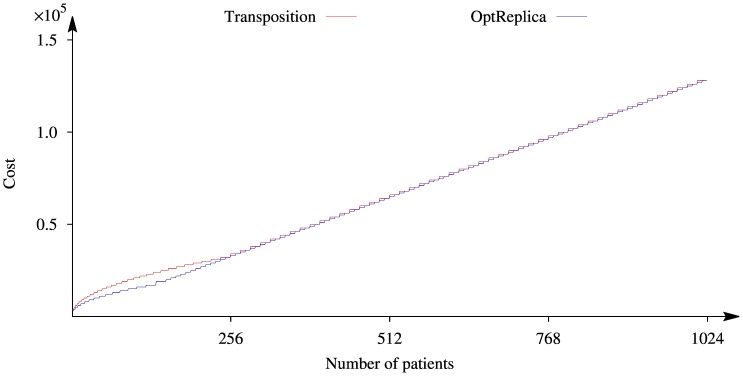
Comparison of cost of sequencing using Transposition and OptReplica strategies with 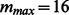.

The comparison of the total cost of the pooled sequencing with replication using Transposition and OptReplica strategies is presented in Figure 7. The figure shows the total cost, obtained using Transposition strategy is higher than the cost, obtained using OptReplica strategy, however a difference appears when the number of patients is small. Both curves have refractive points where curves start rising much faster. These points correspond to the number of patients whose optimal allocation requires to assign more patients in a pool than it is allowed by 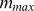. Therefore, it is necessary to increase the number of pools, thus increasing the number of NGS tests.

### Solution of the Multi-objective Optimization Problem

The following experiment is aimed at solution of multi-objective optimization problem, where the probability to find the mutation using larger pools is simultaneously considered. The computations are performed for 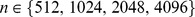 patients, considering the extreme values 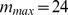 and 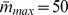, which are determined using previously developed model for the evaluation of the probability to detect a mutation in pooled experiments, when the number of patients increases [11]. The fixed error rate 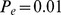 is used as it is a realistic approximation of what is found in this kind of experiments. Since the number of short reads affected by error is independent from the number of patients, in the worst case the signal produced by a SNP can be lost in the noise produced by the experiment. These values are realistic approximations, obtained using real data of pooled experiments.

**Figure 8:**
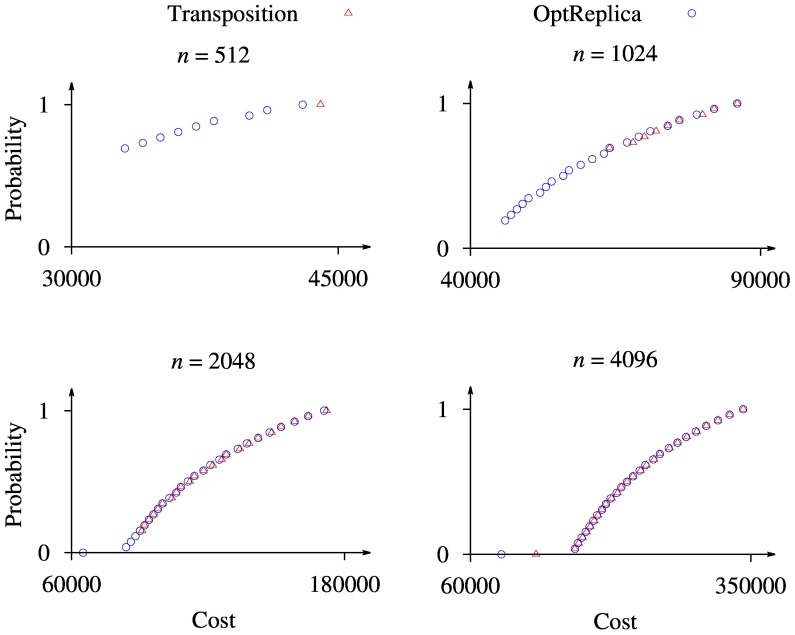
Illustration of Pareto fronts obtained by the complete enumeration with different values of 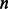.

Pareto fronts, obtained by the complete enumeration of values of 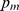 and 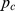 for Transposition strategy and 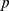 for OptReplica, are illustrated in Figure 8, where the horizontal axis corresponds to the cost of the sequencing, the vertical one to the probability of detecting the mutations, and different plots to different number of patients. One can see from the figure that the Pareto front consists of a single point using Transposition strategy, and several points using OptReplica strategy when 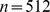. It is natural since all 512 patients can be allocated into such a number 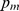 of main pools and a number 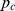 of control pools so that the cost of NGS test would be minimal and without violation of the maximum pool size (e.g. 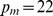 and 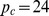). Meanwhile the optimal number of pools for OptReplica strategy is 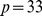 (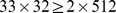), which corresponds to 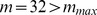. Therefore, a trade off is possible between increasing the number of pools thus increasing the total cost, or exceeding 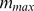, thus decreasing the probability to find the mutations.

When the number of patients is larger, the size of the Pareto front increases, although the Pareto fronts obtained using OptReplica strategy are larger in all cases. Moreover, the cost obtained using OptReplica strategy is always not greater than the cost obtained using Transposition strategy considering the fixed probability. Therefore we can conclude that usage of OptReplica strategy leads to a better solution of the formulated multi-objective optimization problem, comparing to Transposition strategy.

## Discussion

Since the costs of NGS experiments change over time, we decided not to use a currency unit, such as dollar or euro, but rather a ratio of costs between low and high throughput technologies. We fixed a cost of 1000 for one pooled NGS experiment and 8 for each Sanger sequencing.

### In case of no replica, small pools decrease costs

In case of experiments without replica, all found mutations need to be assigned to patients present in the pool. As shown in equation (3), the number of Sanger sequencing increases quadratically with the number of pooled individuals, and it is proportional to the number of expected mutations. Therefore, even if the cost of NGS versus Sanger sequencing is 1000:8, it is better to decrease the size of each pool, rather than increasing the number of patients in each pool. In the present setting, the optimal number of patients per pool is 5 or 6.

### Replication of pools decreases costs

When replicated pools are used, the situation is just the opposite with respect to unreplicated pooling: the experiments are less expensive when the number of patients per pool increases. In Figure 6, the only case in which pooling is more expensive that Optimal pooling without replica, is when there are only four patients per pool. Increasing this number, the reduction of costs is significant, and makes the pooling strategy competitive with respect to unreplicated pools. In both Figures 5 and 6, we report the costs of unreplicated experiments with optimal allocation of patients per pool (Opt. pooling). When comparing the two strategies, it is evident that replicating pools is always the best option. Both Transposition and OptReplica achieve similar results, although in some cases the latter achieves lower costs.

Grouping of patients can reduce the number pools, but Sanger tests must be additionally used to solve the indecision between patients in an identified group. Threfore, grouping can reduce costs only when the total number of patients is small.

### Trade-off between costs and detection probability

When the total budget for sequencing is limited, multi-objective optimization might provide useful information regarding the plan of the experiment. With the proposed model, it is possible to predict the probability to detect mutations, given the number of patients and the costs. In some cases, the difference between the available budget and the required budget might marginally affect the probability to successfully detect mutations. For example, in case of 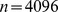 patients (Figure 8), the total cost of all experiments is 342000. If the available budget is only 316000, then the probability to detect mutation is still 0.92, which might be acceptable.

## Conclusions

In this work we propose and study novel strategies for planning the NGS experiments of a large number of patients. We provide optimal configurations of the experiments, in terms of sequencing costs. We develop two different replica strategies, both providing lower overall costs. Finally, we investigate the solution to this problem when not only the cost, but also the probability to detect a mutation, is taken into account.
